# Compartmental anatomical classification of traumatic abdominal injuries from the academic point of view and its potential clinical implication

**DOI:** 10.1186/1752-2897-8-14

**Published:** 2014-09-15

**Authors:** Ayman El-Menyar, Husham Abdelrahman, Hassan Al-Thani, Ahmad Zarour, Ashok Parchani, Ruben Peralta, Rifat Latifi

**Affiliations:** 1 Clinical Research Unit, Section of Trauma Surgery, Hamad General Hospital, Doha, Qatar; 2 Clinical Medicine, Weill Cornell Medical School, Doha, Qatar; 3 Section of Trauma Surgery, Hamad General Hospital, Doha, Qatar; 4 Department of Surgery, University of Arizona, Tucson, AZ, USA; 5 Internal Medicine, Ahmed Maher teaching Hospital, Cairo, Egypt

**Keywords:** Trauma, Abdominal injury, Anatomical compartment, Mortality

## Abstract

**Background:**

The mechanism and outcome of traumatic abdominal injury (TAI) varies worldwide. Moreover, data comparing TAIs in each abdominal compartment are lacking. We aimed to assess from the academic point of view, TAI based on its anatomical compartments.

**Patients & methods:**

We conducted a retrospective study for TAI patients between 2008 and 2011 in Qatar. Patients were categorized according to the involved anatomical compartment (C): intrathoracic (ITC), retroperitoneal (RPC), true abdomen (TAC), and pelvic abdomen (PAC) group. Chi Square test, One-Way ANOVA and multivariate regression analysis were appropriately performed.

**Results:**

Of 6,888 patients admitted to the trauma unit, 1,036 (15%) had TAI that were grouped as ITC (65%), RPC (15%), TAC (13%), and PAC (7%). The mean age was lowest in ITC (29 ± 13) and highest in TAC (34 ± 11) group, (P = 0.001). Motor vehicle crash was the main mechanism of injury in all groups except for PAC, in which fall dominated. Vast majority of expatriates had PAC and TAC injuries. The main abdominal injuries included liver (35%; ITC), spleen (32%; ITC) and kidneys (18%; RPC). Extra-abdominal injuries involved the head in RPC and ITC, lung in ITC and RPC and extremities in PAC. Mean ISS was higher in RPC and ITC. Abdominal AIS was higher in TAC injuries. Overall hospital mortality was 10%: RPC (15%), TAC (11%), ITC (9.4%) and PAC (1.5%). Concurrent traumatic brain injury (OR 5.3; P = 0.001) and need for blood transfusion (OR 3.03; P = 0.003) were the main independent predictors of mortality.

**Conclusion:**

In addition to its academic value, the anatomical approach of TAI would be a complementary tool for better understanding and prediction of the pattern and outcome of TAI. This would be possible if further research find accurate, early diagnostic tool for this anatomical classification.

## Introduction

Trauma is a leading cause of hospitalization, long-term disabilities and mortality worldwide [1]. Traumatic abdominal injury (TAI), whether blunt or penetrating trauma is the third common type of injury (10%) after head (30%) and chest (20%) [2]. In respect to the mechanism of injury (MOI), Motor Vehicle Crash (MVC) is a common cause of admission to the emergency department, accounts for 50-75% of TAI cases [1,3,4]. Moreover, the mechanism and outcome of TAI varies from country to country depending upon the socioeconomic status and culture [5]. In Europe, blunt TAI is more frequent than the penetrating TAI [6]. However, the true frequency of blunt TAI as well as its incidence among out-of-hospital deaths is not well-known. In spite of using advanced imaging and invasive procedures, blunt TAI remains a diagnostic and therapeutic challenge in clinical practice [2,7]. Therefore, adopting a stepwise approach in the evaluation of TAI patients is important to initiate efficient management. Hence, the classic initial step is to consider the mechanism of injury and the vector of force to predict the most likely injury pattern to be assessed [2]. This step should be followed by simultaneous physical examination and bedside imaging. Herein, we hypothesized that in addition to these steps, approaching TAI differently in relation to the abdominal compartments may add new insights for the diagnosis and risk stratification. There are four arbitrary anatomical compartments of the abdomen such as intrathoracic abdomen (ITC) (or upper abdomen), retroperitoneal abdomen (RPC), true abdomen (TAC) (or central abdomen), and pelvic abdomen (PAC) [8]. As data describing the epidemiology of TAI in each abdominal compartment is lacking worldwide, we thought to study the mechanism, patterns and outcome of TAI in relation to the four arbitrary abdominal compartments which have not been reported yet.

## Patients and methods

### Data source

Data were driven retrospectively from the registry database in the section of trauma surgery at Hamad General Hospital (HGH) between January 2008 and December 2011. HGH is a tertiary hospital with the only level I trauma center in the state of Qatar. Qatar is a small rapidly developing country in the Middle East that attracts large number of workers (Expatriates) for construction work. These expatriates represent around 80% of the total population in Qatar.

### Study design and case selection

This is an observational retrospective study. All patients presented with abdominal trauma requiring admission were included in the study. Patients who died at the scene or declared dead on arrival to the Trauma Resuscitation Unit (TRU) were excluded because of incomplete data.

### Exposure variables

The diagnosis of abdominal injury was based on clinical history, physical examination and radiologic imaging in patients who sustained blunt or penetrating injury. Abdominal trauma involved injury to any of the 4 arbitrary anatomical compartments of the abdomen such as intrathoracic abdomen (ITC), retroperitoneal abdomen (RPC), true abdomen (TAC), and pelvic abdomen (PAC) group. This classification was based mainly on the operative findings obtained retrospectively in addition to the imaging tools such as CT scanning. We excluded the obvious overlapped compartment. Also patients who died early before confirming the anatomical compartment were excluded.

On admission, all patients underwent clinical assessment and resuscitation according to Advanced Trauma Life Support (ATLS) guidelines. Patients with unstable pelvic injury were managed by early intervention with activation of massive blood transfusion. Patient data included age, sex, nationality (nationals vs. expatriates), mechanism of injury (MOI), radiological imaging, serum lactate, Injury Severity Scores (ISS), abbreviated injury scale (AIS), abdominal and extra abdominal injuries. Rapid interventions during hospital course were recorded such as intubation, exploratory laparotomy, and open reduction and internal fixation (ORIF) for bone injuries. Intensive care unit and hospital length of stay, need for blood transfusion and hospital mortality were also analyzed.

### Statistical analysis

Patients were divided into 4 groups (GPs) according to the injured abdominal compartment. Data were presented as proportions, median and range, or mean ± SD as appropriate. Differences in categorical variables between age groups were analyzed using the chi square test. The continuous variables were analyzed using one-way ANOVA. For skewed continuous data non-parametric test was performed. Unadjusted predictors for hospital mortality were performed. Two-tailed P values of < 0.05 were considered significant.

### Adjustment for confounding factors

The multivariate logistic regression analysis was performed for the predictors of in-hospital mortality after adjusting the potential covariates that showed significant difference among the study groups (age, ISS, abdominal AIS, retroperitoneal hematoma, use of abdominal CT, traumatic brain injury (TBI), lung injury, blood transfusion, and serum lactate). Adjusted odds ratios, with accompanying 95% confidence intervals, were reported for the respective groups. All data analyses were carried out using the Statistical Package for Social Sciences version 18 (SPSS Inc. USA).

### Ethical statement

This was a retrospective study with no direct contact with the patients and data was collected from the database registry in the trauma unit. All the patients’ information were anonymous and confidential and collected in accordance with the GCP. The study was approved by the medical research center (IRB#12057/12) at Hamad Medical Corporation, Qatar.

## Results

During the period from January 2008 to December 2011, 6,888 cases were admitted to the section of trauma at HGH; of them 1036 (15%) cases had abdominal injuries with mean age of 30.6 ± 13 years and median of 29(1–84) years. Males represented the majority of patients (93%). Details for the 4 intra-abdominal compartments injuries were available for 927 patients. Expatriates (84%) were the most injured population in comparison to the nationals (16%). Table 1 demonstrates the main characteristics in the 2 groups.

**Table 1:** Overall clinical profiles of abdominal injury nationals and expatriates

	**Nationals (16%)**	**Expatriates (84%)**	**P value**
**Age (mean ± SD)**	26 ± 15	32 ± 12	0.001
**Male (%)**	92	93	0.54
**Traffic-related (%)**	79	53	0.001 for all
**Fall from height (%)**	6	29	
**Fall of heavy objects (%)**	0.6	8	
**Head injury (%)**	30	23	0.03
**Lung injury (%)**	33	24	0.01
**Cardiac injury (%)**	1.8	0.9	0.31
**Intrathoracic AC (%)**	77	63	0.001 for all
**Retroperitoneal AC (%)**	14	15	
**True abdomen AC (%)**	7	13	
**Pelvic AC (%)**	1.3	9	
**ISS mean ± SD**	18 ± 11	17 ± 10	0.43
**HLOS (median, range)**	6 (1–134)	8 (1–410)	0.03
**ICU (median, range)**	3 (1–39)	3 (1–150)	0.48
**Blood transfusion (%)**	14	20	0.09
**Abdominal AIS (median, range)**	2 (2–5)	2 (1–5)	0.04
**Mortality (%)**	8.5	8.1	0.86

AC = abdominal compartment.

**Figure 1:**
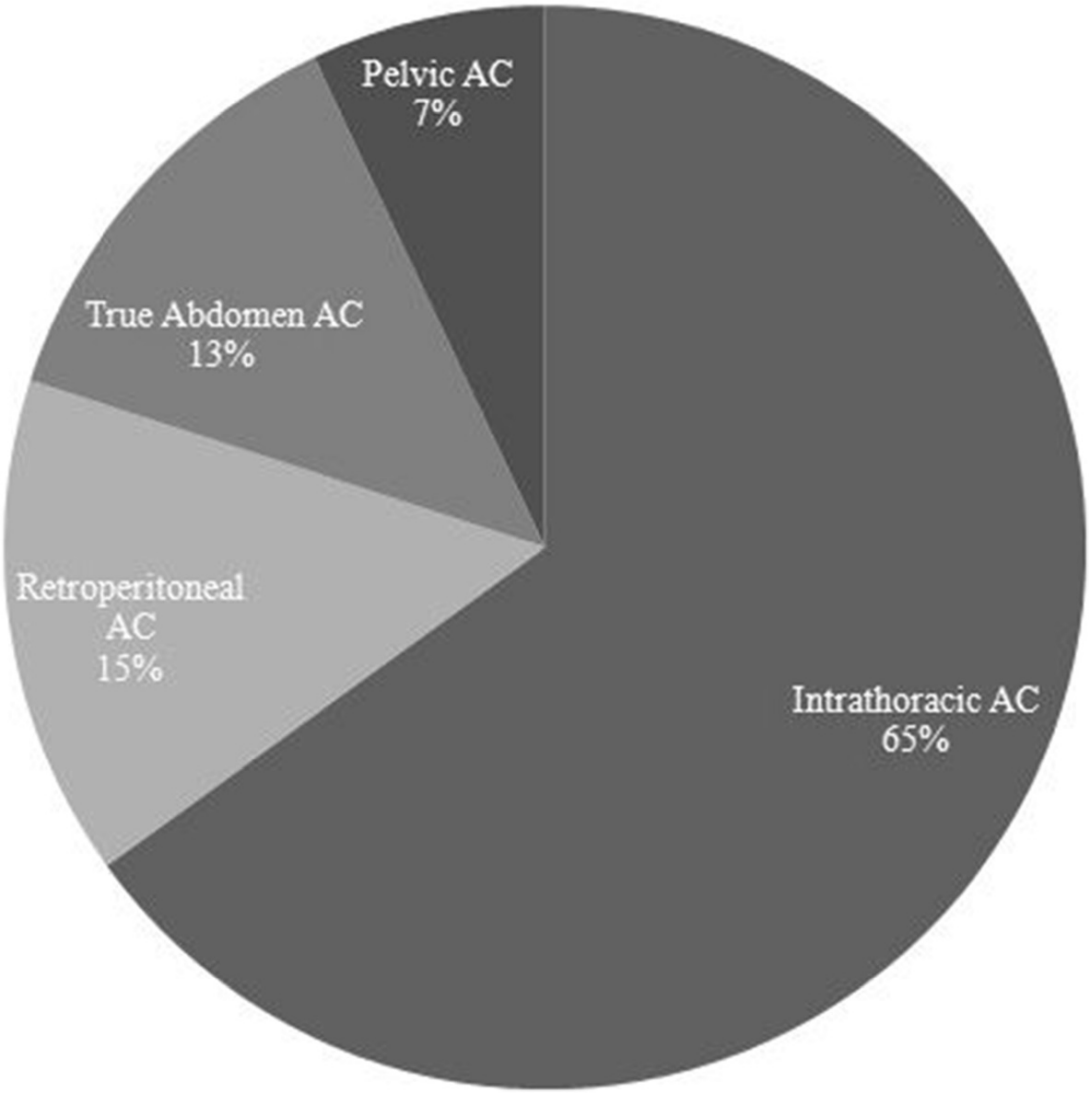
Breakdown of traumatic abdominal injuries according to anatomical compartments.

**Figure 2:**
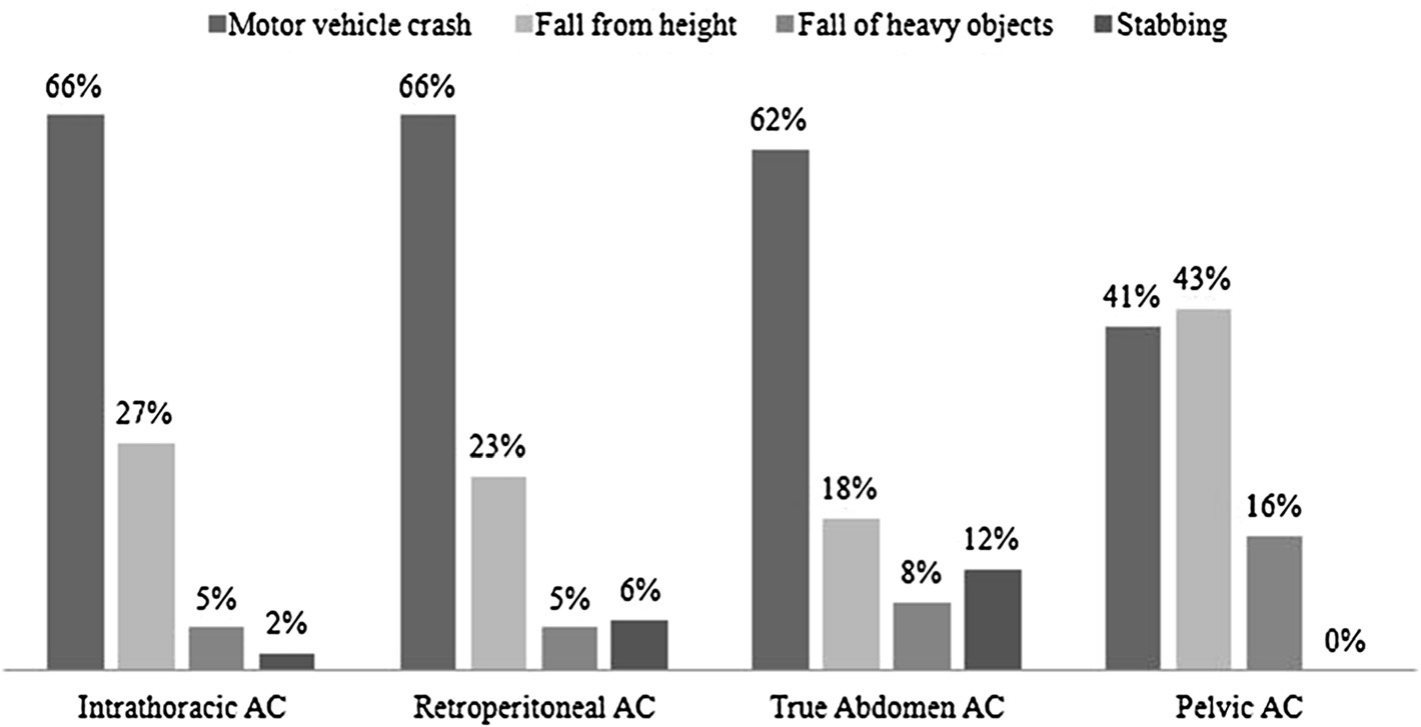
Anatomical abdominal compartments based on the mechanism of injury.

The classification of abdominal injuries based on the four arbitrary anatomical compartments is shown in Figure 1. The vast majority of injuries were depicted in the ITC (65%). Blunt trauma (95%) was the most common MOI in the entire study population. Figure 2 shows the anatomical abdominal compartments based on the mechanism of injury. MVC was the main MOI in the all groups except for PAC, in which fall dominated. Table 2 defines the 4 intra-abdominal compartments according to method of diagnosis, organs involved and mechanism of injury. Tables 3 and 4 summarize the overall descriptive profiles of the study population, in addition to the pattern, mechanism and outcomes of injuries in each compartment.

**Table 2:** Definition of the four arbitrarily anatomical Abdominal compartments

	**Intrathoracic**	**Retroperitoneal**	**True abdomen**	**Pelvic abdomen**
**Organs involved**	Diaphragm (2%)	Kidneys (18%)	SI (12.3%) and LI (5%)	UB (4%)
	Liver (35%)	Ureter (0.2%)	±UB (if full)	Rectum (1.5%)
	Spleen (32%)	Pancreas (3.7%)	± Uterus(if gravid)	Urethra (3%)
	Stomach (1.4%)	Aorta (1%)		Uterus (0.7%)
		Vena cava (0.7%)		
**Diagnosis**	Inaccessible to clinical examination and needs imaging	Very difficult, unless need CT, Angiography, IVP	Significant abdominal physical signs.	Difficult (extraperitoneal)
			X-ray may help if free air +,FAST, ±Diagnostic Peritoneal Lavage	
**Mechanism of injuries**				
**Deceleration**	Hepatic laceration, vascular pedicles	Renal arteries	Mesenteric tear	Splanchnic
**Crushing effect**	Spleen and liver	Kidneys		
**External compression**	Hollow viscous(stomach)		Hollow viscous	

Compartments: All data given in parenthesis as percent were collected from the present study, UB: urinary bladder; CT: Computed Tomography; IVP: Intravenous Pyelogram, SI: small intestine, LI: large intestine.

**Table 3:** Analysis of mechanism, pattern and outcomes of traumatic abdominal injury

	**Overall**	**ITC**	**RPC**	**TAC**	**PAC**	**P value**
**Patients (%)**	927	604 (65)	138(15)	117(13)	68(7)	0.001
**Age (mean ± SD)**	30.6 ± 13	29.2 ± 13	30 ± 12	34.4 ± 11	34.2 ± 13	0.001
**Males (%)**	93	92	92	97	96	0.29
**Expatriates (%)**	84	61	69	75	81	0.001
Mechanisms of injury						
**Blunt (%)**	95	96	95	86	94	0.001
**MVC (%)**	57	61	61	58	39	0.001 for all
**Fall from height (%)**	25	24	21	16	40	
**Stab wound (%)**	3.3	2	5	11	0.0	
**Fall of HO (%)**	7%	5	4	8	15	
Associated injuries						
**Head injury (%)**	24	28	31	11	10	0.001
**Lung injury (%)**	25	33	22	11	8	0.001
**Rib fracture (%)**	32	41	30	10	10	0.001
**Hemothorax (%)**	10	13	11	3	5	0.009
**Retrop hematoma (%)**	7.7	9	7	13	12	0.41
**Pelvic hematoma (%)**	9	3	5	3	13	0.001
**Cardiac injury (%)**	1.1	2	0.7	0.0	0.0	0.31
**ISS (mean ± SD)**	18 ± 10	18.7 ± 10	18.7 ± 12	15 ± 10	13 ± 7	0.001
**Abdominal AIS (median, range)**	2(1–5)	2(1–5)	2(2–5)	3(2–5)	2(2–4)	0.002
**Head AIS (median, range)**	3(1–5)	3(1–5)	3(2–5)	3(2–5)	1(0–4)	0.001

HO = heavy object, ER = emergency room; MVC: Motor vehicle crash; HO: heavy object; ISS: Injury Severity score; AIS: Abbreviated injury score.

**Table 4:** Interventions and complications

	**Overall**	**ITC**	**RPC**	**TAC**	**PAC**	**P value**
**Interventions**						
**Expl lap (%)**	27	20	20	84	27	0.001
**Spinal surgery (%)**	1.2	1	0	1.7	1.5	0.53
**ORIF (%)**	20	19	20	15	21	0.66
**Intubation (%)**	27	28	28	31	19	0.041
**CT abdomen (%)**	90	91	93	76	82	0.001
**Serum lactate(mean ± SD)**	3.8 ± 2.6	3.7 ± 2.5	3.7 ± 2.6	4 ± 3	3.7 ± 1.9	0.69
**Blood transfusion (%)**	20	16	23	23	28	0.02
**Wound infection (%)**	4	1.8	3.6	10.3	1.5	0.001
**Sepsis (%)**	0.8	0.3	0	2.6	1.5	0.02
**Admission on arrival**						0.001 for all
**Operating room (%)**	25	19	19	69	30	
**TICU (%)**	41	45	40	15	34	
**Surgical wards (%)**	34	35	40	15	36	
**Died in ER (%)**	0.2	0.3	0	0	0	
**Intensive care stay (median, range)**	3 (1–150)	3(1–94)	3.5(1–64)	3.5(1–34)	3(1–150)	0.76
**Length of stay (median, range)**	8 (1–261)	6(1–261)	9(1–118)	7(1–80)	18(1–80)	0.001
**Mortality (%)**	10	9.4	15	11	1.5	0.02

Expl lap: exploratory laparotomy; ORIF: Open Reduction Internal Fixation; MBT: Massive blood transfusion; TICU: Trauma intensive care unit; ER: emergency room; Retrop: retroperitoneal; CT: computed Tomography.

(1) Intrathoracic abdominal compartment (ITC) constituted the majority of cases who sustained TAI (65%). Patients in this group were younger in comparison to other groups with a mean age of 29.2 ± 13 years (p = 0.001). The MOI was mainly related to MVC and in a rate similar to RPC group (61%), P = 0.001. In comparison to other compartments, ITC was highly associated with rib fractures (41%), lung injury (33%) and hemothorax (13%). TBI was reported in 28% of patients. Patients in this group also had higher ISS (18.7 ± 10). ITC patients were more frequently admitted to TICU (45%) in comparison to other patients (p = 0.001). The only two (0.3%) patients who died in this study population in the emergency room belonged to this group.

(2) Retroperitoneal abdominal compartment (15%): In comparison to other groups, patients in the RPC group were more frequently screened by abdomen CT scanning (93%), P = 0.001. Also, the mean ISS (18.7 ± 12, P = 0.001) and rate of associated TBI (31%, p = 0.001) were the highest in this group. Rib fractures (30%), lung contusion (22%) and Hemothorax (11%) ranked the second common associated injuries after ITC. Next to ITC, 40% of RPC patients were admitted directly from trauma room to the ICU.

(3) True abdomen compartment (13%): Patients in TAC group were older in comparison to other groups with a mean age of 34 ± 11 yrs (p = 0.001) and 75% of them were expatriates. In this group, the incidence of MVC was 58%, whereas stabbing (11%) was higher in comparison to other groups, (P = 0.001). Abdominal AIS (median 3 and range 2–5), retroperitoneal hematoma (13%), need for intubation (31%), and rate of wound infection (10.3%) were higher in this group when compared to others. Nearly two thirds of these patients were admitted directly from trauma room to operating theater (p = 0.001). Exploratory laparotomy (84%) was performed frequently in this group (p = 0.001).

(4) Pelvic abdominal compartment (7%): Patients in PAC group were mainly expatriates (81%). Fall from height (40%) and fall of heavy objects (15%) were more evident MOI in this group when compared to other groups, (p = 0.001). Most of patients (36%) were admitted directly to the surgical wards. Patients in this group showed the least incidence of TBI (10%), lowest serum lactate levels (2.7 ± 1.9) and least mean ISS (13 ± 7). Pelvic hematoma (13%) and extremities injury requiring ORIF (21%) were more frequent in PAC patients. The need for transfusion (28%) was higher among these patients. The most prolonged hospital stay in our study population was reported in this group (p = 0.001).

### Outcome and multivariate analysis

**Figure 3:**
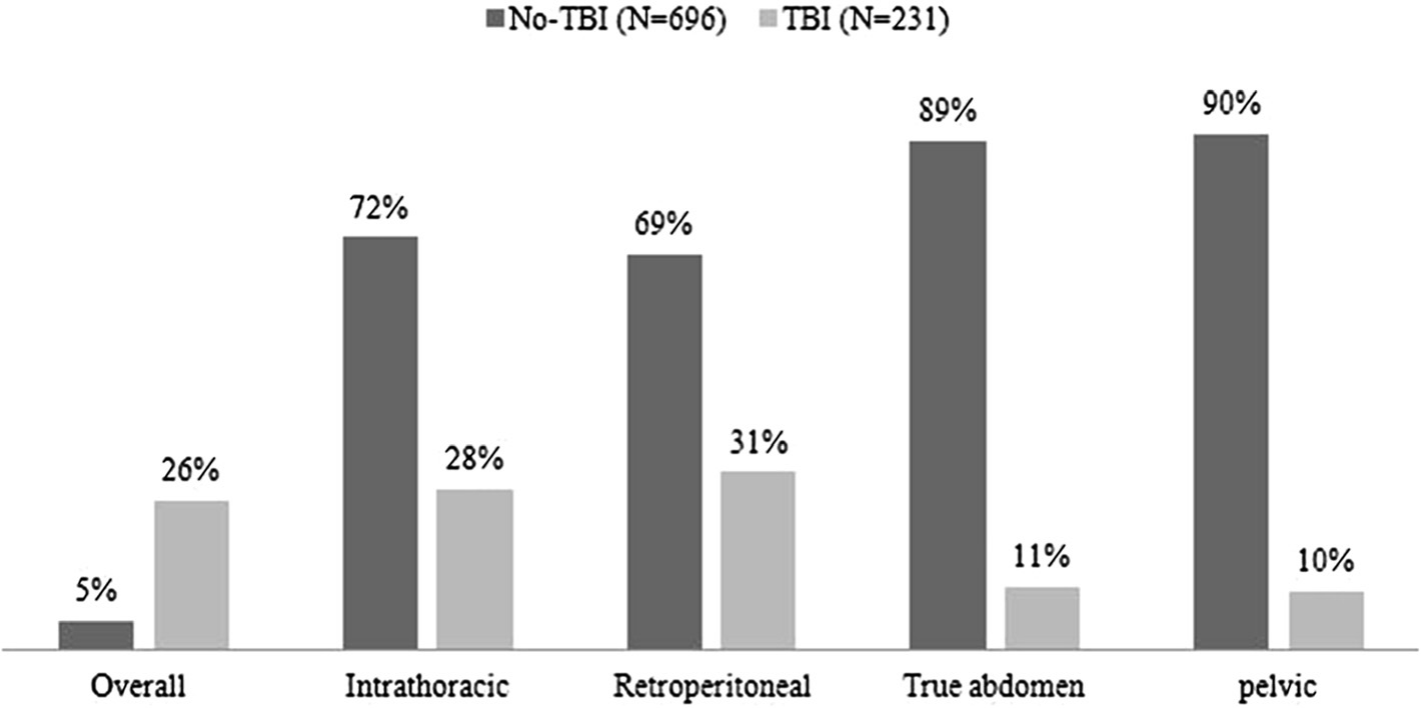
Mortality in different compartments based on the presence of concurrent traumatic brain injury (TBI).

The overall mortality rate was 10% in abdominal trauma patients: RPC (15%), TAC (11%), ITC (9.4%) and PAC (1.5%), p = 0.02. Concurrent TBI increased mortality 5 folds in comparison to absence of TBI. Figure 3 demonstrates the mortality in different abdominal compartments based on the presence of concurrent traumatic brain injury. Also, the unadjusted in-hospital mortality was significantly higher in those who presented with abdominal AIS ≥3 (n = 362) as compared to patients with median abdominal AIS < 3 (n = 681); (15% vs. 7%; p = 0.001). After adjustment for relevant co-variates (age, head and lung injury, retroperitoneal hematoma, ISS, Abdominal AIS, serum lactate, need for transfusion, exploratory laparotomy procedure, and abdominal CT scanning), multivariate logistic regression analysis showed that TBI (OR 5.3; 95% CI 2.17-13.3), need for blood transfusion (OR 3.03; 95% CI 1.45-6.35), mean ISS (OR 1.1; 95% CI 1.07-1.17), age (OR 1.03; 95% CI 1.00-1.05), and serum lactate (OR 1.03; 95% CI 1.00-1.06) were the independent predictors of mortality among TAI patients (Table 5).

**Table 5:** Predictors of mortality

	**OR (95% confidence interval)**	**P value**
Age	1.03 (1.00 - 1.05)	0.03
Injury severity score	1.1 (1.07 - 1.17)	0.001
Abdominal AIS	0.8 (0.48 - 1.18)	0.23
Lung injury	1.3 (0.62 - 2.78)	0.47
Traumatic brain injury	5.3 (2.17 - 13.3)	0.001
Retroperitoneal hematoma	1.3 (0.52 - 3.15)	0.58
Abdominal CT scan	0.43 (0.13 - 1.4)	0.16
Serum lactate	1.03 (1.00 - 1.06)	0.04
Need for Blood transfusion	3.03 (1.45 - 6.35)	0.003

## Discussion

Up to the best of our knowledge, description of the TAI in relation to the four arbitrary anatomical compartments of the abdomen is not known. This anatomical approach may help better understanding of the pattern and outcome of TAI. Classically, TAI has been tackled from different aspects including mechanism of injury (blunt vs. penetrating), organ function (solid vs. hollow) or peritoneum-organ relationship. Yet, the injury classification according to anatomical proximity of organs has not been addressed. Moreover, the peculiar association between anatomical compartments and variables such as age, MOI, pattern of injury, associated extra-abdominal injuries and outcomes is not well-defined.

In the current study, majority of patients who sustained abdominal injuries were young in their most productive age and were predominantly males. Similar findings were also reported by earlier studies [5,9]. The most common mechanism of injury in this study was blunt trauma (95%), this finding puts our study as the largest series with 1,036 TAI cases which exceeds the earlier published report on blunt TAI in the literature [9-11]. Moreover, the MOI explains the higher incidence of associated extra-abdominal injury in our analysis. This high incidence of blunt abdominal injuries could be attributed to high frequency of MVCs and falls from height (mainly workplace-related injuries). Furthermore, MVC was the commonest mechanism of injury among all the injured compartments except pelvic abdominal compartment (PAC) injury, in which fall from height dominated. Expatriates in Qatar constitute around 80% of the population; this explains the involvement of large number of expatriates (84%) in our study. Vast majority of expatriates had PAC and TAC injuries. The main abdominal injuries included liver (35%; ITC), spleen (32%; ITC) and kidneys (18%; RPC). Hollow viscus injuries were identified as small and large bowel injures (12% and 5% respectively) in TAC and urinary bladder injury (4%) in PAC injuries. The rate of hematomas (pelvic and retroperitoneal) was higher in PAC and TAC injuries. The main extra-abdominal injuries involved the head in RPC and ITC (31% and 28%, respectively), lung and heart in ITC (33% and 2%, respectively) and RPC (22% and 0.7%, respectively). The rate of extremity injuries was observed in 21% in PAC injuries. Although blood loss is a common complication with solid organ injuries (i.e., ITC and RPC), our study demonstrated paradoxically that the need for transfusion was higher in TAC and PAC injuries. This finding could be explained in part by the higher rate of surgery (i.e., Exploratory Laparotomy, long bone, and spinal fixation) and associated retroperitoneal and pelvic hematomas in these compartments. Hospital length of stay was prolonged among PAC patients, a finding that could be related to higher incidence of extremities injuries, higher rate of surgery, sepsis and lower mortality.

The overall mortality rate, in the current analysis, was 10%; the peak mortality was seen in patients with RPC and TAC (15% and 11%) followed by 9.4% in ITC injuries, while the lowest rate was 1.5% in patients with PAC injury.

In the present study, associated TBI was reported in a quarter of the cases and its presence was associated with 5 fold increase in the mortality rate. The median head AIS was highest in TAC and RPC patients and matched up their high mortalities. Our finding is consistent with previous data which demonstrated TBI to be the most frequent cause of mortality in patients with blunt trauma [12]. Moreover, after adjustment for relevant co-variates the associated TBI was a strong independent predictor of mortality in our cohort (adjusted OR = 5.3). In addition, the need for blood transfusion was also an important independent predictor for mortality, showing a 3 fold increased risk of mortality (adjusted OR = 3.0). The multivariate logistic regression analysis also demonstrated other predictors of mortality in TAI such as age (OR 1.03; p = 0.03), mean ISS (OR 1.1; p = 0.001), and serum lactate (OR 1.03; p = 0.04). Serum lactate level was higher in patients with TAC in comparison to other compartments.

An Indian study by Lone et al. [13] reported 144 TAI cases (53% penetrating and 47% blunt trauma) and found that the mortality rates were 9.2% and 8.2% among patients with penetrating and blunt injuries, respectively. The cause of death was mainly related to hemorrhagic shock. Gad et al. [9] analyzed TAI in 248 Egyptian cases (69% blunt and 31% penetrating) and reported 26% overall mortality rate in terms of 58% in penetrating and 12% in blunt trauma . The higher mortality rate was due to delay in treatment, a factor that was not measured in our study. Lund et al. [14] from Denmark reported higher mortality (up to 56%) in hemodynamically unstable patients with abdominal injuries undergoing emergency laparotomy. Smith et al. [11] from Australia studied 1,224 TAI cases (79% were blunt injury) over 8 yrs and reported an overall mortality rate between 7-9%. Our findings showed that the mortality rate increased from 7% to reach 15% in those who presented with higher abdominal AIS. Particularly, the median abdominal AIS was higher in TAC injuries compared to other compartments (P = 0.001). However, abdominal AIS was not identified as an independent predictor of mortality in our study.

### Limitations

Retrospective nature of the study is one of the limitations. The data obtained from the registry did not perfectly include detailed operative findings and information regarding brought-in-dead patients. The anatomical compartment classification is arbitrary that may miss some overlapping organs; this can be overcome through further studies. The study tried to demonstrate that whether compartmentalization of the injuries inside the abdomen has, in addition to the academic value, clinical significance that clinicians and students of trauma surgery need to recognize. To this end, we believe that not all intra-abdominal organ injuries are the same and carry different clinical implication. We understand that we are still far from considering this methodology as a standard clinical practice. However, this study sets a step forward focusing on organ injuries based on the anatomical compartment concept. This step should utilize thorough clinical examination and advanced imaging tools. Moreover, this concept will have diagnostic as well as management implications. For instance, recognizing that injuries within TAC carries higher chance of surgical rather than conservative management will have significant implications on the resources and time management in a busy trauma centers. Perhaps our next study should combine anatomical compartments with organ injury severity score of American Association of Surgery of Trauma (AAST). This may lead to a new scoring system, useful to the clinicians around the world.

## Conclusion

Traumatic abdominal injury is a common clinical challenge that varies according to the mechanism and site (abdominal region) of injury. The anatomical approach of TAI may be of academic interest as a re-visit of the basic medicine. Also, it could be a complementary tool for better understanding and diagnosis of TAI and therefore further studies are needed to outline these compartments preoperatively.

## Competing interests

The authors declare that they have no competing interests.

## Authors’ contributions

AE carried out study design, data collection, analysis, and interpretation and drafted the manuscript. HAR carried out data collection and interpretation and drafted the manuscript. HA carried out data collection, and interpretation and drafted the manuscript. AZ carried out data collection, and interpretation and drafted the manuscript. AP carried out data collection and interpretation and drafted the manuscript. RP carried out data collection, and interpretation and drafted the manuscript. RL carried out data analysis, and interpretation and drafted the manuscript. “All authors read and approved the final manuscript.
